# Piperine-Loaded Nanoparticles: In Vitro Evaluation of a Botanical Nanoixodicide Against *Rhipicephalus microplus* and *Amblyomma mixtum* Larvae Resistant to Conventional Treatments

**DOI:** 10.3390/pharmaceutics18070828

**Published:** 2026-07-07

**Authors:** Romario García-Ponce, José Pablo Villarreal-Villarreal, Rocío Álvarez-Román, Adriana E. Flores, María Julia Verde-Star, José Ezequiel Viveros-Valdez, David Mizael Ortiz-Martínez, David Gilberto García-Hernández, Sergio Arturo Galindo-Rodríguez

**Affiliations:** 1 Laboratorio de Nanotecnología, Facultad de Ciencias Biológicas, Universidad Autónoma de Nuevo León, San Nicolás de los Garza 66455, Mexico; romario.garciapo@uanl.edu.mx (R.G.-P.); maria.verdest@uanl.edu.mx (M.J.V.-S.); jose.viverosvld@uanl.edu.mx (J.E.V.-V.); dortizm@uanl.edu.mx (D.M.O.-M.); david.garciahrz@uanl.edu.mx (D.G.G.-H.); 2 Facultad de Medicina Veterinaria y Zootecnia, Universidad Autónoma de Nuevo León, General Escobedo 66054, Mexico; pablo.villarrealvl@uanl.edu.mx; 3 Departamento de Química Analítica, Facultad de Medicina, Universidad Autónoma de Nuevo León, Monterrey 64460, Mexico; rocio.alvarezrm@uanl.edu.mx; 4 Laboratorio de Entomología Médica, Facultad de Ciencias Biológicas, Universidad Autónoma de Nuevo León, San Nicolás de los Garza 66455, Mexico; adriana.floressr@uanl.edu.mx

**Keywords:** polymeric nanoparticles, piperine, ixodicide, *Rhipicephalus microplus*, *Amblyomma mixtum*

## Abstract

**Background/Objectives**: Tick infestations represent a major economic problem for livestock farming due to the hematophagous nature of these parasites and their crucial role as vectors of pathogens. The prolonged use of synthetic chemical ixodicides has led to resistance, prompting the search for alternative tick-control strategies. In this study, piperine-loaded polymeric nanoparticles were successfully developed using Eudragit^®^ L100-55 (NP_Pip_1) and Eudragit^®^ RLPO (NP_Pip_2) polymers, which were physicochemical characterized and evaluated against *Rhipicephalus microplus* and *Amblyomma mixtum* larvae resistant to conventional treatments. **Methods/Results**: NP_Pip_1 and NP_Pip_2 exhibited mean particle sizes of 107.30 ± 6.34 nm and 101.00 ± 1.51 nm, polydispersity index (PDI) values of 0.08 ± 0.02 and 0.05 ± 0.03, ζ potentials of −5.78 ± 2.14 and 32.7 ± 0.74 mV and encapsulation percentages (%E) of 17.44 ± 1.56 and 1.43 ± 0.11. The encapsulation efficiencies (%EE) were 69.76 ± 6.25% and 37.40 ± 2.89%, respectively. Free piperine showed LC_50_ and LC_90_ values of 1822.25 and 5981.74 µg/mL against *R. microplus*, and 1904.18 and 6213.28 µg/mL against *A. mixtum*. In contrast, NP_Pip_1 reduced LC_50_ and LC_90_ values by 1.83- and 2.2-fold against *R. microplus* and by 1.61- and 1.44-fold against *A. mixtum*, indicating improved ixodicidal activity compared with free piperine. NP_Pip_2 showed smaller reductions in LC_50_ and LC_90_ of 1.34- and 1.33-fold against *R. microplus* and 1.43- and 1.41-fold against *A. mixtum*. **Conclusions**: These results demonstrate that incorporating piperine into polymeric nanoparticles significantly improves its ixodicidal activity against resistant tick larvae, positioning this platform as a promising strategy for the development of alternative treatments against ticks resistant to conventional treatments.

## 1. Introduction

Ticks represent a significant problem for bovine production systems [1]. Their impact is attributed to their hematophagous feeding behavior, which causes substantial productive losses including weight reduction, anemia, skin damage, decreased reproductive efficiency, and reduced milk yield in cattle. Moreover, they serve as vectors for pathogens responsible for diseases such as babesiosis and anaplasmosis [2,3].

*Rhipicephalus microplus* and *Amblyomma mixtum* are tick species with a high economic impact on livestock systems in Mexico [2,4]. Estimates suggest that annual economic losses caused by *R. microplus* alone amount to US$ 573.61 million in Mexico [5]. *A. mixtum* is recognized as the second most common tick species parasitizing cattle in the region. Although the economic losses associated with this species have not yet been calculated, its presence significantly affects livestock-raising regions [4,6,7].

For decades, tick control has relied almost exclusively on the use of chemical ixodicides such as organophosphates, pyrethroids, amitraz, fipronil, and combinations of these compounds. However, their intensive and inappropriate application has promoted the emergence and rapid spread of tick populations resistant to commercially available products. Additionally, these practices have led to negative consequences, including environmental contamination, toxic effects on non-target organisms, residues in food products and health risks to humans [8,9,10,11]. Consequently, there is a critical need to seek alternative approaches for managing tick infestations.

The use of plants and their secondary metabolites for therapeutic and pest-control purposes in human and animal health is widely practiced worldwide [12,13,14]. Several studies have shown that these natural compounds possess acaricidal activity, and they are considered safer alternatives with lower risks to human health and the environment [14,15]. Furthermore, they are characterized by their low toxicity towards non-target organisms and their potential to mitigate the development of resistance in arthropod populations [16,17]. Despite their effectiveness against ticks, current research has not successfully translated these findings into commercial products due to the difficulties associated with the formulation of plant compounds, the elimination of organic solvents, and the challenge of designing vehicles capable of protecting them against environmental factors [17,18,19].

Piperine is an alkaloid found in the fruits of different plant species of the genus Piper [20]. This naturally occurring compound has demonstrated multiple biological activities, including antioxidant, antitumor, analgesic, anti-inflammatory, hepatoprotective, and antimicrobial effects [21,22]. Furthermore, fruit extracts of *Piper tuberculatum* and *Piper nigrum* with high piperine content have shown ixodicidal activity against larvae and engorged adult females of *R. microplus* [23,24]. More recently, da Silva et al. [25] reported the in vitro activity of piperine against *R. microplus* larvae, demonstrating its potential as a treatment for tick infestations. However, its low aqueous solubility (LogP = 3.69) remains a major limiting factor for its biological application in cattle.

In this context, the formulation of botanical compounds with biological activity using polymeric nanoparticles can effectively overcome the problem of low aqueous solubility, eliminating the need to use organic solvents [26]. These nanoparticles are colloidal systems designed for drug delivery, whose development has made it possible to address and treat various agricultural and public health pests [27,28,29,30,31,32]. Their ability to encapsulate and transport active ingredients, combined with a high surface area due to their nanometric size, enhances biological efficacy and may improve the availability of the active compound at the target site [33]. Currently, polymeric nanoparticles represent a good alternative for the formulation and delivery of natural products with insecticidal and acaricidal activities [27,28,34]. Eudragit^®^ L100-55 and Eudragit^®^ RLPO are pharmaceutical-grade methacrylate copolymers that have been widely employed in topical nanoformulations because of their biocompatibility, physicochemical stability, and ability to encapsulate compounds that are poorly soluble in water [35,36,37]. Eudragit^®^ L100-55 is an anionic polymer containing carboxylic acid groups that impart a negative surface charge, whereas Eudragit^®^ RLPO contains quaternary ammonium groups that confer a positive surface charge [38]. Several studies have demonstrated the utility of these polymers in topical nanoformulations designed to improve the stability, dispersion, and delivery of hydrophobic bioactive compounds across biological surfaces [35,36,37,39,40,41]. These characteristics make them attractive candidates for the encapsulation of piperine, a poorly water-soluble alkaloid whose biological activity may benefit from enhanced dispersion and increased contact with the target organism. Furthermore, differences in the surface charge of Eudragit^®^ L100-55 and Eudragit^®^ RLPO may influence their interactions with the external structures of ticks and, consequently, the transport of the encapsulated compound. However, further studies are required to elucidate the mechanisms involved.

Considering the established ixodicidal activity of piperine, this study aimed to develop, characterize, and evaluate the in vitro ixodicidal activity of piperine-loaded polymeric nanoparticles prepared using Eudragit^®^ L100-55 and Eudragit^®^ RLPO. The nanoformulations were evaluated against larvae of *R. microplus* and *A. mixtum* resistant to conventional acaricides.

## 2. Materials and Methods

### 2.1. Materials

Piperine, cypermethrin, and chlorpyrifos were purchased from Sigma-Aldrich^®^ (St. Louis, MO, USA). The commercial product used, Bovitraz^®^ (12.5% amitraz), was purchased from Elanco (Zapopan, Mexico). Fipronil was obtained from Toronto Research Chemical Inc (TRC) (Toronto, ON, Canada). Eudragit^®^ L 100-55 and Eudragit^®^ RLPO polymers were kindly donated by Helm (Naucalpan, Mexico).

### 2.2. Preparation and Characterization of Polymeric Nanoparticles

Piperine-loaded nanoparticles were prepared using the nanoprecipitation method as previously described [42]. Briefly, 5 mL of an organic phase containing the polymer (65 mg of Eudragit^®^ L100-55 or 500 mg of Eudragit^®^ RLPO, depending on the formulation) and 15 mg of piperine were dissolved in a solvent mixture composed of acetone, isopropanol, and methanol (2:2:1, *v*/*v*/*v*). The organic phase was then injected into 10 mL of an aqueous phase containing 0.5% (*w*/*v*) polyvinyl alcohol, maintained at 25 ± 2 °C under moderate magnetic stirring. The diffusion-driven mixing of the organic and aqueous phases reduced polymer solubility, causing its aggregation and the subsequent formation of nanoparticles. Subsequently, the organic solvents were removed using a rotary evaporator (Laborota 4003 control, Heidolph Instruments, Schwabach, Germany) until a final volume of 10 mL was reached. Two nanoparticle formulations corresponding to the Eudragit^®^ L 100-55 (NP_Pip_1) and Eudragit^®^ RLPO (NP_Pip_2) polymers were obtained. Blank nanoparticles (NP_Bco_1 for Eudragit^®^ L 100-55 polymer and NP_Bco_2 for Eudragit^®^ RLPO) were generated using an identical preparation protocol, except for the omission of piperine.

#### 2.2.1. Size, Polydispersity Index (PDI) and ζ Potential (ζP)

Nanoparticle size distribution and polydispersity index (PDI) were evaluated by dynamic light scattering (DLS) using a Zetasizer Nano-ZS90 (Malvern Instruments, Worcestershire, UK) with measurements performed at a 90° detection angle. Surface charge characteristics were determined through laser Doppler electrophoresis, and the results were expressed as zeta potential (ζP).

#### 2.2.2. Stability of Nanoparticles

The physical stability of the nanoformulations was evaluated during 180 days of storage under controlled conditions (25 ± 2 °C and 47 ± 2% relative humidity). At predetermined time intervals, particle size, polydispersity index (PDI), and zeta potential (ζ) were determined. The values obtained on day 180 were compared with those recorded on day 0 using Student’s *t*-test. Differences were considered statistically significant at *p* < 0.05.

#### 2.2.3. Fourier Transform Infrared (FT-IR) Analysis

The analysis of Eudragit^®^ L100-55 and Eudragit^®^ RLPO polymers, piperine, non-piperine NPs (NP_Bco_1, NP_Bco_2) and piperine-loaded nanoparticles (NP_Pip_1 and NP_Pip_2) was performed by FT-IR spectroscopy. Analyses of potential molecular interactions were performed with 64 scans in the range of 4000 to 400 cm^−1^ using an Optical Frontier FT-IR spectrometer (PerkinElmer, Waltham, MA, USA).

#### 2.2.4. Quantification of Piperine Incorporated into Nanoparticles

An analytical method using high-performance liquid chromatography (HPLC) was developed to quantify the piperine incorporated into the nanoparticles. A Varian HPLC system with a UV-Vis detector (Varian Co., Palo Alto, CA, USA) and a C18 reversed-phase column (ZORBAX, Eclipse XDB, 2.1 × 150 mm, 5 μm, Agilent Technologies^®^, Santa Clara, CA, USA) was used. The mobile phase consisted of milliQ water with formic acid (0.1% *v*/*v*) and methanol in a 30:70 ratio, with a flow rate of 0.3 mL/min at 30 °C. A calibration curve was constructed using five concentration levels ranging from 5 to 80 μg/mL. Standard piperine solutions were prepared in a mixture of Milli-Q water containing 0.1% (*v*/*v*) formic acid and methanol (30:70, *v*/*v*), and detection was performed at 343 nm. As part of a preliminary analytical assessment of the method, linearity (R^2^), limit of detection (LOD), limit of quantification (LOQ), and precision were determined according to the recommendations of the Eurachem Guide [43].

To determine the amount of piperine associated with the nanoparticles, the nanoparticle suspensions were subjected to centrifugation at 33,600× *g* for 60 min using an Allegra 64R centrifuge (Beckman Coulter Inc., Indianapolis, IN, USA). The sediment was subsequently dissolved in the mobile phase and injected into the HPLC. Each assay was performed in triplicate. Finally, the encapsulation percentage (%E) and encapsulation efficiency percentage (%EE) were determined using Formulas (1) and (2).

%E = (mg of piperine in nanoparticles/mg of the polymer + mg of piperine in organic phase) × 100(1)

% EE = (mg of encapsulated piperine/mg of piperine in organic phase) × 100(2)

### 2.3. Ticks

Field-collected engorged female ticks of *R. microplus* and *A. mixtum* were recovered from cattle naturally infested with ticks and not subjected to acaricide treatments. Sampling was conducted at a production unit in Tantoyuca, Veracruz, Mexico (21°21′07.6″ N, 98°3′35.29″ W). Ticks were manually removed and placed in plastic vials for transport to the laboratory, where their taxonomic identification was carried out using the keys of Dantas-Torres et al. [44] and Guzman-Cornejo et al. [45].

#### 2.3.1. Establishment of Tick Populations in the Laboratory

Colonies of *R. microplus* and *A. mixtum* were established and maintained at the Multidisciplinary Research Laboratory of the Faculty of Veterinary Medicine and Zootechnics, Universidad Autónoma de Nuevo León (Mexico). Engorged females were rinsed, dried with absorbent paper, and incubated at 28 ± 2 °C with 80–90% relative humidity to promote oviposition. The resulting egg masses were transferred to incubation tubes and maintained under identical environmental conditions until hatching. Larvae between 7 and 14 days of age were selected for subsequent resistance diagnosis and bioassays.

#### 2.3.2. Resistance Diagnosis

Resistance to conventional ixodicides amitraz, cypermethrin, chlorpyrifos, and fipronil was diagnosed using the DD reported by Martínez-Ibáñez et al. [46]. To evaluate resistance to amitraz, the commercial product Bovitraz^®^, which contains the active compound in a 12.5% (*w*/*v*) emulsifiable concentrate formulation, was used by the modified larval immersion test (LIT) [47], at a DD (0.0002%, *v*/*v*). Resistance to cypermethrin and chlorpyrifos was evaluated by the larval package test (LPT), at DD of 0.5% (*w*/*v*) and 0.3% (*w*/*v*), respectively. For fipronil, the modified LIT [48] was performed with a DD of 0.05% (*w*/*v*). All tests were carried out in triplicate, counting live and dead larvae, considering those capable of walking as live and those incapable of walking as dead [47].

#### 2.3.3. Bioassays with Piperine-Loaded Nanoparticles

The ixodicidal activity of piperine against *R. microplus* and *A. mixtum* larvae was evaluated using the LIT [49] modified by Castro-Janer et al. [48]. Briefly, 1 mL of piperine solutions at concentrations of 375, 500, 750, 1000, 2000, 4000, 6000, and 7000 μg/mL were dispensed into 2 mL microcentrifuge tubes. Approximately 500 larvae were exposed to each treatment by immersion for 10 min, during which the tubes were gently agitated by hand to ensure uniform contact with the solution. After exposure, the excess liquid was removed, and the treated larvae were distributed into filter paper packets containing approximately 100–200 individuals each. The packets were incubated at 28 ± 2 °C and 80–90% relative humidity for 24 h. Following incubation, larval mortality was determined by counting the number of live and dead specimens.

Piperine-loaded nanoparticles were concentrated using a rotary evaporator (Control Laborota 4003, Heidolph Instruments, Schwabach, Germany) and *R. microplus* and *A. mixtum* larvae were tested using the same assay at piperine concentrations of 140, 280, 560, 1120, 2240, 3360, 4480 and 5600 μg/mL for NP_Pip_1 and 150, 300, 600, 1200, 2400, 3600, 4800 and 6000 μg/mL for NP_Pip_2. Larval mortality was corrected according to the FAO recommendations using the Abbott formula [50] (Formula (3)). If mortality in the control group exceeded 5%, the bioassay test was considered invalid and repeated. The 50 and 90% lethal concentrations (LC_50_ and LC_90_) were determined from the mortality results.

M = [(T − C)/(100 − C)] × 100(3)

where T represents the mortality observed in the treatment group and C represents the mortality observed in the control group.

### 2.4. Statistical Analysis

Larval mortality data obtained at the evaluated concentrations were subjected to Probit analysis with Polo-Plus software (LeOra Software^®^ Inc., Berkeley, CA, USA) to estimate LC_50_ and LC_90_ values and their 95% confidence intervals. Lethal concentration estimates were considered significantly different when the associated confidence intervals did not intersect.

## 3. Results

### 3.1. Preparation and Characterization of Nanoparticles

Piperine-loaded nanoparticles were successfully prepared with Eudragit^®^ L100-55 (NP_Pip_1) and Eudragit^®^ RLPO (NP_Pip_2) polymers, yielding mean particle sizes of 107.30 ± 6.34 nm for NP_Pip_1 and 101.00 ± 1.51 nm for NP_Pip_2 (Table 1). Nanoparticles without piperine (NP_Bco_1 and NP_Bco_2) exhibited smaller sizes than the loaded nanoparticles. The polydispersity index (PDI) was less than 0.2 in all cases. Nanoparticles prepared with Eudragit^®^ L100-55 presented a ζP of −5.78 ± 2.14 mV, whereas Eudragit^®^ RLPO exhibited a potential of 32.70 ± 0.74 mV (Table 1).

### 3.2. Quantification of Piperine Incorporated into Nanoparticles

The high-performance liquid chromatography method used to quantify piperine incorporated into the nanoparticles showed a correlation coefficient (R^2^) of 0.99, an LOD of 1.36 μg/mL, an LOQ of 4.13 μg/mL, and a precision of 7.20%. The calibration curve exhibited a linear relationship described by the equation y = 2,000,000x + 915,220 within the concentration range studied. The resulting encapsulation percentage (%E) and encapsulation efficiency (%EE) values of the formulations are summarized in Table 1.

### 3.3. Stability of Nanoparticles

**Figure 1:**
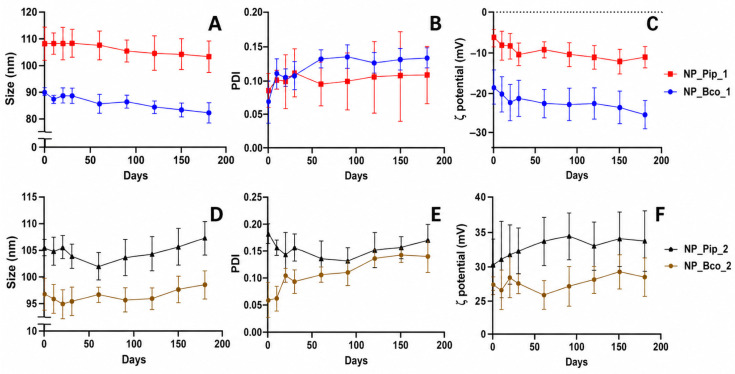
Stability of nanoparticle formulations over 180 days. (**A**) Particle size, (**B**) PDI and (**C**) ζ potential of NP_Pip_1 and NP_Bco_1. (**D**) Particle size, (**E**) PDI and (**F**) ζ potential of NP_Pip_2 and NP_Bco_2 (mean ± SD, n = 3).

The size, polydispersity index (PDI), and ζP parameters of the piperine-loaded nanoformulations (NP_Pip_1 and NP_Pip_2) and piperine-free nanoparticles (NP_Bco_1 and NP_Bco_2) were monitored over a 180-day period (Figure 1). No significant changes were observed in these three parameters during this period.

### 3.4. Fourier Transform Infrared Spectroscopy (FT-IR)

**Figure 2:**
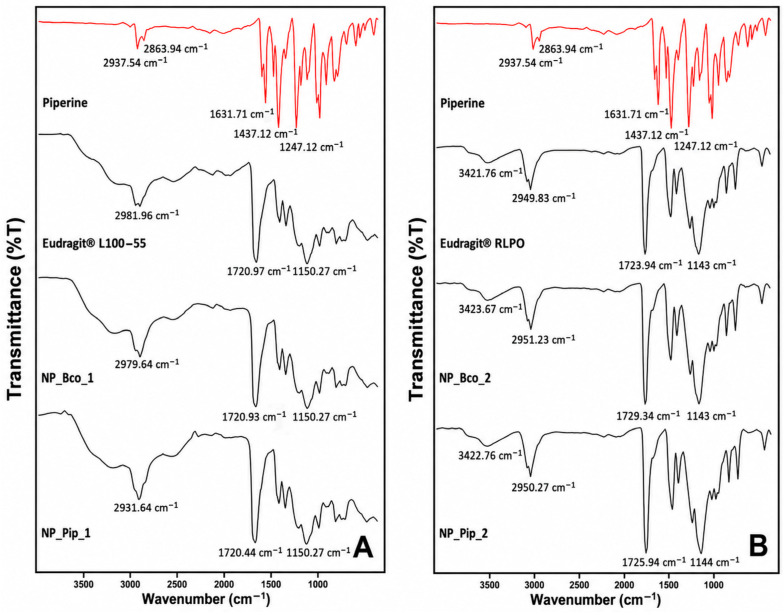
FT-IR spectra of piperine, polymers, and nanoparticles without piperine and with piperine. (**A**) Formulations with Eudragit^®^ L100-55, and (**B**) Formulations with Eudragit^®^ RLPO.

FT-IR spectra of piperine-loaded nanoparticles (NP_Pip_1 and NP_Pip_2) were recorded and compared with those of pure piperine, Eudragit^®^ L100-55 and RLPO polymers, and blank nanoparticles (NP_Bco_1 and NP_Bco_2). This qualitative analysis was performed to evaluate the preservation of the characteristic functional groups of the formulation components and to identify potential spectral changes associated with the nanoencapsulation process. In addition, the comparison of the spectra allowed the assessment of whether new absorption bands indicative of covalent bond formation resulting from chemical reactions between piperine, and the polymeric matrices were generated during nanoparticle preparation (Figure 2).

### 3.5. Diagnosis of Resistance

Resistance diagnosis revealed that the *R. microplus* and *A. mixtum* larval populations exhibited varied mortality rates when exposed to the discriminating doses (DD) evaluated for each ixodicide [46]. Larvae showing mortality below 100% at the DD of the acaricide were classified as resistant, while those with 100% mortality were considered susceptible (Table 2). Both tick species demonstrated resistance to cypermethrin and amitraz, but remained susceptible to fipronil. Regarding the organophosphate chlorpyrifos, the *R. microplus* population was found to be susceptible, whereas the *A. mixtum* population was confirmed to be resistant.

### 3.6. Ixodicidal Activity of Piperine-Loaded Nanoparticles

As detailed in Table 3, the ixodicidal activity of free piperine against larvae resistant to synthetic ixodicides exhibited LC_50_ = 1822.25 μg/mL and LC_90_ = 5981.74 μg/mL values for *R. microplus*, while for *A. mixtum* the values were 1904.18 μg/mL and 6213.28 μg/mL, respectively. The NP_Pip_1 formulation achieved an LC_50_ of 990.96 μg/mL and LC_90_ of 2713.23 μg/mL in *R. microplus* larvae. In *A. mixtum* larvae, the same formulation exhibited an LC_50_ of 1180.78 μg/mL and an LC_90_ of 4296.39 μg/mL. Likewise, the NP_Pip_2 formulation presented an LC_50_ of 1353.45 μg/mL and an LC_90_ of 4481.12 μg/mL in *R. microplus* larvae, and an LC_50_ of 1330.15 μg/mL and an LC_90_ of 4397.50 μg/mL in *A. mixtum*. The formulations NP_Bco_1 and NP_Bco_2, used as negative controls, did not exhibit ixodicidal activity in either tick species. Overall, these findings indicate that incorporation of piperine into polymeric nanoparticles enhances its ixodicidal activity compared to the free compound.

## 4. Discussion

Tick control is one of the major challenges for livestock producers worldwide. Conventional control methods have several limitations, highlighting the need to research and develop new therapeutic agents, novel delivery vehicles, and innovative application methods to address these limitations more effectively. The use of botanical extracts and their compounds as ixodicidal agents represents an alternative, given their generally favorable safety profile and low environmental impact [51,52,53]. However, their application is limited by poor aqueous solubility and inadequate stability under environmental conditions. Piperine is an alkaloid that has garnered significant interest due to its diverse biological properties [22], including insecticidal [54] and ixodicidal activities [25]. Limitations to its application are related to its lipophilic nature (LogP = 3.69) [55], which limits its aqueous solubility.

The use of polymeric nanoparticles offers an effective approach to overcoming solubility limitations of plant-derived compounds, functioning as carriers that ensure optimal delivery of the active compound [56]. However, despite the potential advantages of these nanometric systems for ixodicidal application treatments, control of formulation parameters is required to ensure their efficacy [34].

In this study, piperine-loaded nanoparticles were prepared by the nanoprecipitation technique using the anionic copolymer Eudragit^®^ L100-55 and the cationic copolymer Eudragit^®^ RLPO. The mean particle size of NP_Pip_1 and NP_Pip_2 formulations was less than 110 nm (Table 1). This is consistent with sizes reported in other studies where piperine was incorporated into Eudragit^®^ S100 and Eudragit^®^ E100 nanoparticles, with dimensions of 130 and 187 nm, respectively [34,57]. Furthermore, the size of 104 nm reported by Politi et al. [58] for piperine-loaded Eudragit^®^ S100 nanoparticles is very similar to the average sizes obtained in this study. The nanometric size of polymeric nanoparticles facilitates interaction through physical contact, ingestion and inhalation, as described by Shahzad and Manzoor [59]. Furthermore, this parameter may play an important role in the delivery of ixodicidal compounds, as nanoparticles within the nanometric size range could promote a closer interaction with the tick tegument. Considering that exocuticular pores with diameters of approximately 100–130 nm has been described in some tick species [60], it can be hypothesized that nanoparticles of similar dimensions may be capable of penetrating or distributing through these structures. On the other hand, the PDI reflects the size distribution of nanoparticles, where values close to 0 indicate homogeneous (monodisperse) systems with minimal variability in particle size, whereas values close to 1 indicate a heterogeneous size distribution [61]. In this study, the PDI values were below or close to 0.20 (Table 1), indicating a high homogeneity in the obtained nanoparticles. This size uniformity is a relevant attribute, since it could ensure that the individual interactions of the nanoparticles with the tick integument occur homogeneously. Another physicochemical characteristic evaluated was the ζP, which is directly related to the stability of the nanoparticles in suspension, considering that values higher than +30 mV or lower than −30 mV indicate a greater electrostatic repulsion among the nanoparticles, which can reduce aggregation and improve the stability of the formulations [58]. The ζP values determined for formulations NP_Pip_1 and NP_Pip_2 were −5.78 mV and 32.70 mV, respectively. The value for formulation NP_Pip_1 was not close to 30 mV, which could be assumed to present instability [62]; however, the formulation did not present significant variations in size, PDI and ζP after 180 days (Figure 1), demonstrating that the formulation was stable over this period. The ζP values in the NP_Pip_1 and NP_Pip_2 formulations showed a difference in charges, this is because an anionic polymer (Eudragit^®^ L 100-55) and a cationic one (Eudragit^®^ RLPO) were used. This feature is important because it could facilitate the interactions and internalization of polymeric nanoparticles through the membranes of ticks.

The encapsulation percentage (%E) is defined as the fraction of active compound incorporated into 100 parts of the nanoparticle. In this study, nanoformulations NP_Pip_1 and NP_Pip_2 showed %E values of 17.44% and 1.43%, respectively. This marked difference between the two could be related to the physical characteristics of the formulations, particularly the polymer: piperine ratio, with ratios of 4:1 for NP_Pip_1 and 33:1 for NP_Pip_2. This difference suggests that, due to the high polymer content present, the incorporation of piperine into the matrix could be limited, which would explain the lower encapsulation percentage observed in the formulation prepared with Eudragit^®^ RLPO (NP_Pip_2).

**Figure 3:**
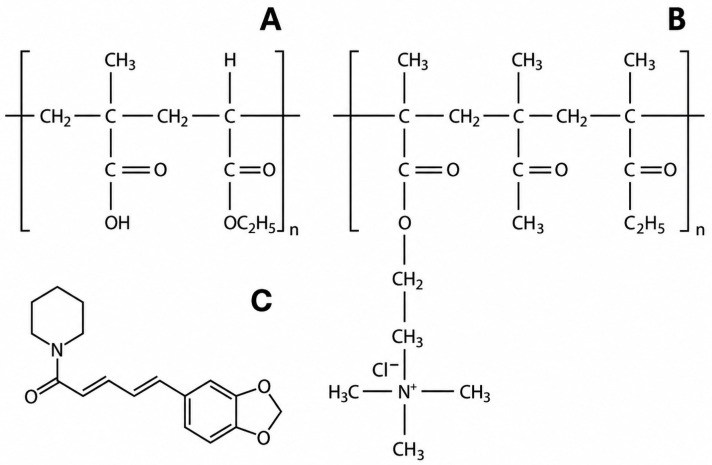
Monomeric structures of the polymers and piperine. (**A**) Eudragit^®^ L100-55, (**B**) Eudragit^®^ RLPO, and (**C**) Piperine.

On the other hand, encapsulation efficiency (%EE) is the percentage of the active compound incorporated into the nanoparticles relative to the total amount added during their preparation. In this study, formulations NP_Pip_1 and NP_Pip_2 presented %EE values of 69.76% and 37.40%, respectively (Table 1). These results show notable differences in %EE values between the two formulations, which can be explained by the structural differences between the Eudragit^®^ L100-55 and Eudragit^®^ RLPO polymers (Figure 3). Eudragit^®^ L100-55 is an anionic copolymer whose structure and lower permeability could favor greater incorporation and retention of hydrophobic compounds such as piperine, which could explain the higher encapsulation efficiency observed in NP_Pip_1. In contrast, Eudragit^®^ RLPO is a cationic copolymer with quaternary ammonium groups and a more permeable matrix, which could lead to a less compact association of the active compound within the nanoparticle and contribute to the differences observed in the %EE of NP_Pip_2. Compared to other studies, the %EE values obtained were lower than those reported by de Oliveira et al. [34], who achieved a piperine %EE of 98.20% in Eudragit^®^ S100 nanoparticles. However, although the authors used the nanoprecipitation technique to obtain nanoparticles, their formulation conditions differed, specifically the use of a lipid component (i.e., caprylic/capric acid triglycerides) to solubilize the piperine and generate nanocapsules with an internal lipid phase surrounded by a polymer shell. This lipid phase may provide a more favorable environment for the incorporation of lipophilic molecules, which likely contributed to the high %EE achieved in that study. Furthermore, the %EE results obtained from NP_Pip_1 were consistent with those reported by Politi et al. [58], who formulated piperine-loaded Eudragit^®^ S100 nanoparticles, achieving an EE of 76.2%. This coincidence could be attributed to the similarities between the Eudragit^®^ L100-55 and Eudragit^®^ S100 polymers [38], both of which are composed of methacrylic acid and ethyl acrylate in Eudragit^®^ L100-55 and methyl methacrylate in Eudragit^®^ S100.

In relation to the FT-IR analysis, the spectrum corresponding to piperine (Figure 2A,B) showed characteristic bands that include signals at 2937.54 cm^−1^ corresponding to the aromatic C–H stretching, 2863.94 cm^−1^ aliphatic C–H stretching of the methylenedioxy group, 1631.71 cm^−1^ C=O stretching, 1437.12 cm^−1^ aromatic C=C stretching in the benzene ring and 1247.12 cm^−1^ asymmetric =C–O–C stretching, which coincide with those previously reported by Stasiłowicz et al. [63] and Ren et al. [57]. On the other hand, in the spectrum of the Eudragit^®^ L100-55 polymer, important signals from the functional groups present in the monomeric unit of this copolymer derived from methacrylic acid and ethyl acrylate were identified (Figure 2A). The C–H stretching band of the alkyl groups was observed at 2981.96 cm^−1^. The presence of the carbonyl group (C=O) produces a signal at 1720.97 cm^−1^ corresponding to the C=O stretching of the carboxylic acid and the ester. Finally, the signal at 1150.27 cm^−1^ corresponds to the C–O stretching of the ester. These signals coincide with those reported by Solís-Cruz et al. [64]. Similarly, the spectrum of the Eudragit^®^ RLPO polymer presented characteristic signals of its functional groups (Figure 2B). The bands at 3421.76 cm^−1^ showed the presence of tertiary amino groups, in addition to bands at 1723.94 and 1143 cm^−1^ corresponding to the C=O and C–O bonds of the ester groups, respectively. These signals agreed with the spectra reported by Singh et al. [65] and Mehta et al. [66]. Finally, the spectra of the NP_Bco_1 and NP_Pip_1 formulations showed a great similarity with the spectrum of the Eudragit^®^ L100-55 polymer (Figure 2A), while the spectra of NP_Bco_2 and NP_Pip_2 (Figure 2B) were very similar to those of the Eudragit^®^ RLPO polymer. This indicates that no significant structural changes or new covalent bonds were formed between piperine and polymers during nanoparticle preparation.

On the other hand, resistance to ixodicides is a global problem that threatens both the livestock economy and the health of humans and animals. This issue significantly hinders effective tick control and prevention. In Mexico, previous studies have documented that tick species such as *R. microplus* and *A. mixtum* have developed resistance to several common acaricides, including coumaphos, chlorpyrifos, amitraz, cypermethrin, and fipronil [11,67,68,69,70,71]. In contrast to these reports, our study observed that the *R. microplus* population was susceptible to chlorpyrifos, while the *A. mixtum* population showed resistance. Furthermore, both species demonstrated susceptibility to fipronil and resistance to amitraz and cypermethrin (Table 2). These results indicate that the populations of *R. microplus* and *A. mixtum* used in this study were resistant to amitraz and cypermethrin, whereas only the *A. mixtum* population was resistant to chlorpyrifos.

The activity of free piperine was determined against *R. microplus* and *A. mixtum* larvae, yielding LC_50_ values of 1822.25 and 1904.18 µg/mL, respectively (Table 3). This compound has been identified in methanolic extracts of *Piper nigrum* and *Piper longum* as a major component, demonstrating significant effects on the mortality rates of *R. microplus*, with percentages ranging from 12.50 to 95.80% for *P. nigrum* and between 29.20% and 87.50% for *P. longum*, associating its ixodicidal activity with piperine [24]. Similarly, extracts obtained with hexane, ethyl ether, ethanol, and methanol from *P. tuberculatum* fruits, containing 24% piperine, showed larvicidal activity against *R. microplus* with LC_50_ values between 40 and 4490 µg/mL [23]. Additionally, extracts of *P. nigrum* fruits and stems have been evaluated against *R. australis*, demonstrating 100% larvicidal activity at 50 mg/mL [72]. Furthermore, da Silva et al. [25] recently demonstrated ixodicidal activity of piperine against *R. microplus*, with an LC_50_ of 6.04 mM (1723.50 µg/mL), very similar to the result obtained in this work. Furthermore, these authors demonstrated in vitro and in silico that piperine inhibits the enzyme acetylcholinesterase; they suggest that this is a mechanism of action associated with its toxicity against ticks. On the other hand, previous studies have demonstrated the ixodicidal activity of piperine; however, no reports have evaluated its efficacy against *R. microplus* larvae resistant to cypermethrin and amitraz or *A. mixtum* larvae resistant to chlorpyrifos, cypermethrin, and amitraz (Table 2).

The toxicity of nanoformulations NP_Pip_1 and NP_Pip_2 against *R. microplus* and *A. mixtum* was evidenced by the LC_50_ and LC_90_ values (Table 3). Formulation NP_Pip_1 exhibited an LC_50_ of 990.96 μg/mL and an LC_90_ of 2713.23 μg/mL in larvae of the *R. microplus* population. For the *A. mixtum* larval population, formulation NP_Pip_1 showed an LC_50_ of 1180.78 μg/mL and an LC_90_ of 4296.39 μg/mL. The NP_Pip_2 formulation showed an LC_50_ of 1353.45 μg/mL and an LC_90_ of 4481.12 μg/mL in *R. microplus* larvae. For the *A. mixtum* population, this formulation showed an LC_50_ of 1330.15 μg/mL and an LC_90_ of 4397.50 μg/mL. The ixodicidal activity of piperine had been previously evidenced [23,25]; however, the incorporation of this compound in polymeric nanoparticles allows us to evaluate its activity in an aqueous medium, being of great importance for the application of piperine as an alternative for tick control. Similarly, piperine-free nanoparticles (NP_Bco_1 and NP_Bco_2) were evaluated against *R. microplus* and *A. mixtum* larvae without showing any ixodicidal activity. Therefore, the formulation components are not expected to interfere with the ixodicidal activity of piperine. This result is favorable since nanoparticles without an active ingredient should act as excipients, with no biological effects [73,74].

Piperine-loaded nanoparticles (NP_Pip_1 and NP_Pip_2) showed lower LC_50_ and LC_90_ values than free piperine (Table 3), indicating that lower concentrations were required to induce mortality in *R. microplus* and *A. mixtum* larvae, which indicates that lower concentrations were required to achieve the same biological effect. In *R. microplus*, NP_Pip_1 reduced LC_50_ and LC_90_ by 1.83- and 2.2-fold, respectively, whereas in *A. mixtum* these reductions were 1.61- and 1.44-fold. NP_Pip_2 showed smaller reductions in LC_50_ and LC_90_ of 1.34- and 1.33-fold in *R. microplus*, and 1.43- and 1.41-fold in *A. mixtum*, compared with free piperine. Overall, these results indicate that the incorporation of piperine into polymeric nanoparticles improved its ixodicidal activity. This effect can be attributed to the physicochemical properties of the polymeric nanoparticles, particularly particle size and surface charge. These factors could promote more efficient interaction with the tick’s integument, increasing the contact surface area. In turn, these attributes could facilitate the internalization of nanoparticles and the overcoming of biological barriers. Furthermore, the small size of the nanoparticles could facilitate their penetration through biological barriers, contributing to greater piperine availability and, consequently, to an increase in ixodicidal activity [19,56,75,76,77]. On the other hand, NP_Pip_1 showed lower LC_50_ and LC_90_ values than NP_Pip_2. In *R. microplus*, NP_Pip_1 reduced LC_50_ and LC_90_ by 1.36- and 1.65-fold, respectively, compared with NP_Pip_2, suggesting improved biological activity against tick larvae. In *A. mixtum*, no statistically significant differences were detected between both formulations; however, NP_Pip_1 exhibited slightly lower LC_50_ and LC_90_ values (1.12- and 1.02-fold, respectively) than NP_Pip_2. These differences in ixodicidal activity may be attributed to the physicochemical properties of the polymers used: Eudragit^®^ L100-55 (anionic) in NP_Pip_1 and Eudragit^®^ RLPO (cationic) in NP_Pip_2, which probably interact differentially with the physiological barriers of the tick integument. Furthermore, the higher %E and %EE values obtained with NP_Pip_1 (Table 1) suggest that this formulation retained and was able to deliver a greater amount of piperine, which could explain its greater bioactivity compared to NP_Pip_2. In summary, these results demonstrate that the chemical nature of the carrier polymer, along with the encapsulation characteristics, could be determining factors in the ixodicidal efficacy of the polymeric nanoparticles. However, further studies are required to elucidate the interaction mechanisms between the polymer and the parasite, and release profiles of the active compound will be necessary to confirm these observations.

The development of tick control treatments using nanoparticles as delivery systems for natural products is an emerging area of research, and therefore, only a few studies have been reported so far. Recently, the larvicidal activity against *R. microplus* of mixtures of synthetic ixodicides (i.e., cypermethrin and chlorpyrifos) and terpenes (i.e., citral, menthol and limonene) loaded in solid lipid nanoparticles and nanostructured lipid carriers was evaluated, obtaining LC_50_ values between 3.3 and 5.5 μg/mL. Furthermore, the formulations proved to be stable and demonstrated potential as effective tick control treatments [76]. In addition, Figueiredo et al. [78] incorporated similar mixtures of synthetic ixodicides and terpenes in zein nanoparticles. They evaluated a range of concentrations from 4 to 466 μg/mL of the components in *R. microplus* larvae, obtaining mortality rates greater than 80% at concentrations greater than 29 μg/mL, in addition to efficacy between 40.5 and 60.1% at concentrations of 466 μg/mL in engorged adult females. These investigations demonstrate the potential of nanoparticulate systems for the development of ixodicide treatments, allowing different compounds of botanical origin to be formulated individually or in combination, offering alternatives to contribute to the control of ticks and thereby mitigate resistance to synthetic ixodicides.

This work demonstrated the potential of piperine-loaded nanoformulations for use as a treatment against *R. microplus* and *A. mixtum* larvae. However, further studies are required to assess their field efficacy and to develop safe methodologies for the administration of botanical nanoformulations in animals.

## 5. Conclusions

The present study demonstrated the feasibility of incorporating piperine into polymeric nanoparticles based on Eudragit^®^ L100-55 and Eudragit^®^ RLPO, which exhibited adequate physical stability during the evaluation period. The incorporation of piperine into polymeric nanoparticles, particularly in the NP_Pip_1 formulation, resulted in 1.83- and 2.2-fold reductions in LC_50_ and LC_90_ against *R. microplus*, respectively, and 1.61- and 1.44-fold reductions against *A. mixtum*, demonstrating improved ixodicidal activity relative to free piperine. This improvement may be associated with key characteristics of the nanoformulations, including their reduced particle size, encapsulation efficiency, and physicochemical properties, which could favor interactions with the parasite tegument and improve the availability of the active compound. Overall, the results obtained under in vitro conditions suggest that the incorporation of piperine into polymeric nanoparticles represents a promising strategy for enhancing its ixodicidal activity against *R. microplus* and *A. mixtum* larvae. However, further research is required to optimize the formulations and expand their physicochemical and biological characterization, including the evaluation of their stability in different biological media, release profiles, morphological properties, and efficacy under in vivo and field conditions. In addition, future studies should investigate the mechanisms underlying the enhanced biological activity of these nanoformulations, assess their safety in non-target organisms, and support the development of safe, effective, and environmentally sustainable botanical nanoformulations for tick control.

## Figures and Tables

**Table 1:** Characterization of piperine-loaded polymeric nanoparticles and nanoparticles without piperine (mean ± SD, n = 3).

Formulation	Size (nm)	PDI ^a^	ζ Potential (mV)	%E ^b^	%EE ^c^
NP_Pip_1 ^d^	107.30 ± 6.34	0.08 ± 0.02	−5.78 ± 2.14	17.44 ± 1.56	69.76 ± 6.25
NP_Pip_2 ^e^	101.00 ± 1.51	0.05 ± 0.03	32.70 ± 0.74	1.43 ± 0.11	37.40 ± 2.89
NP_Bco_1 ^f^	89.97 ± 1.32	0.06 ± 0.03	−12.40 ± 4.70	-	-
NP_Bco_2 ^g^	95.50 ± 2.94	0.18 ± 0.01	37.90 ± 2.90	-	-

^a^ PDI: Polydispersity index, ^b^ %E: Encapsulation percentage, ^c^ %EE: Encapsulation efficiency percentage, ^d^ NP_Pip_1: Piperine-loaded Eudragit^®^ L100-55 nanoparticles, ^e^ NP_Pip_2: Piperine-loaded Eudragit^®^ RLPO nanoparticles, ^f^ NP_Bco_1: Eudragit^®^ L100-55 nanoparticles (without piperine), ^g^ NP_Bco_2: Eudragit^®^ RLPO nanoparticles (without piperine).

**Table 2:** Susceptibility and resistance status of *Rhipicephalus microplus* and *Amblyomma mixtum* larvae based on discriminant dose of different acaricides (mean ± SD, *n* = 3).

		*R. microplus*		*A. mixtum*	
Ixodicide	Discriminating Dose (% p/v)	Mortality (%)	Diagnosis	Mortality (%)	Diagnosis
Chlorpyrifos ^a^	0.3	100 ± 0.00	Susceptible	98.23 ± 10.00	Resistant
Cypermethrin ^a^	0.5	43.36 ± 8.00	Resistant	35.34 ± 7.40	Resistant
Amitraz ^b^	0.0002	57.68 ± 17.00	Resistant	39.28 ± 13.33	Resistant
Fipronil ^c^	0.05	100.00 ± 0.00	Susceptible	100.00 ± 0.00	Susceptible
Control	----	00.00 ± 0.00	----	00.00 ± 0.00	----

^a^ Control for cypermethrin and chlorpyrifos was trichloroethylene and olive oil, 2:1 (larval pack test), for ^b^ Amitraz the control was water, and for ^c^ Fipronil the control was 10% acetone solution and 0.04% Triton X-100 (larval immersion test).

**Table 3:** The 50 and 90% lethal concentrations of treatments against *Rhipicephalus microplus* and *Amblyomma mixtum* larvae.

Treatment	LC_50_ (μg/mL)	CI 95%	LC_90_ (μg/mL)	CI 95%
	*R. microplus*			
NP_Pip_1	990.96 ^c^	890.43–1099.51	2713.23 ^b^	2368.58–3183.84
NP_Pip_2	1353.45 ^b^	1215.63–1501.79	4481.12 ^a^	3884.23–5299.53
Piperine	1822.25 ^a^	1578.06–1868.64	5981.74 ^a^	4356.76–6845.17
NP_Bco_1	0.00	-	0.00	-
NP_Bco_2	0.00	-	0.00	-
	*A. mixtum*			
NP_Pip_1	1180.78 ^b^	1057.03–1315.62	4296.39 ^b^	3678.79–5160.61
NP_Pip_2	1330.15 ^b^	1194.95–1475.56	4397.50 ^b^	3819.89–5194.46
Piperine	1904.18 ^a^	1758.57–2058.28	6213.28 ^a^	5501.86–7161.86
NP_Bco_1	0.00	-	0.00	-
NP_Bco_2	0.00	-	0.00	-

^a,b,c^: Different letters indicate significant differences among treatments based on non-overlapping 95% confidence intervals (*p* < 0.05); CI 95%: 95% Confidence intervals.

## Data Availability

The original contributions presented in this study are included in the article. Further inquiries can be directed to the corresponding author.
